# iGraphCTC: an inter-connected graph convolutional network for comprehensive clinical trial collaborations

**DOI:** 10.1038/s41598-026-40836-5

**Published:** 2026-03-02

**Authors:** Jiseon Jang, Hyeongjin Ahn, Eunil Park

**Affiliations:** 1 Samsung E&A, Seoul, 05288 Republic of Korea; 2 Department of Applied Artificial Intelligence, Sungkyunkwan University, Seoul, 03063 Republic of Korea; 3 Department of Computer Science and Engineering, Jaume I University, Castellon, 12071 Spain

**Keywords:** IGraphCTC, Graph network, Clinical trials collaboration, Graph based Recommendation, Chronic disease, Computational science, Scientific data

## Abstract

Pharmaceutical companies are increasingly expanding their global presence by engaging in collaborative clinical research to meet the growing demand for effective chronic disease treatments. However, identifying suitable affiliations and collaboration networks remains a significant challenge. To tackle this, we propose iGraphCTC, a novel framework for clinical trial collaboration that utilizes an adapted Graph Convolutional Network (GCN) to streamline the identification of potential collaborators. The key contribution lies in its ability to integrate multidimensional clinical data (geographical and intervention attributes) into the recommendation process. Based on both geographical and intervention datasets, iGraphCTC achieves maximum improvements of 16.08% (AUC), 14.28% (F1-Score), and 6.68-17.44% (Accuracy@K). These results highlight its capability to enhance recommendation accuracy by addressing limitations of previous models and integrating clinical insights into the recommendation process. Our results demonstrate the effectiveness of graph-oriented approaches in identifying collaborative activities and pinpointing potential collaborators, providing valuable insights into the dynamics of the pharmaceutical industry’s collaborative landscape.

## Introduction

In the rapidly evolving landscape of clinical research, identifying effective collaboration strategies is paramount for advancing medical discoveries and improving patient outcomes. Despite the growing complexity of chronic diseases, existing collaboration models often fail to address the nuanced interactions among various stakeholders involved in clinical trials. This study introduces a novel approach to optimize collaboration networks by leveraging advanced graph-based recommendation systems. Our research specifically targets the enhancement of strategic partnerships among pharmaceutical companies, research institutions, and clinical trial sponsors, thereby addressing the critical challenges of inefficiency and high costs in clinical trial execution. Pharmaceutical companies have actively engaged in clinical research through collaborative networks to expand their global market presence. With the rising prevalence of chronic diseases, such as diabetes and stroke ^1^, several organizations and companies are expected to initiate such clinical trials. However, because of the perceived impracticality and high costs associated with most clinical trials ^2^, these entities have primarily concentrated on identifying suitable collaboration partners and sponsors. Institutions also form collaborative groups to conduct research on global clinical trials. This indicates the importance of prioritizing the exploration of affiliations and collaboration networks ^3^.

Additionally, the challenge of utilizing medical text data stems from the extensive and diverse information^4^. To address these challenges, we propose a novel graph-based recommendation model, named iGraphCTC (inter-connected Graph Convolutional Network for Clinical Trial Collaborations), specifically designed to guide affiliations in chronic disease trials. Using this approach, we can visualize and investigate the clinical relationships and interactions among institutions using graph-oriented approaches. iGraphCTC represents social network interconnections through a graph, with each user (collaborators or sponsors) represented as a node within the graph. The relationships among users are presented as edges, forming a user-user matrix. This method intricately populates a matrix with connected nodes, defining the underlying structure of the network for more effective recommendations. Our proposed approach leverages GCNs to fuse multifaceted clinical data into a robust collaboration prediction system. The primary novelty of iGraphCTC is its capability to incorporate supplementary clinical data–specifically geographical location and intervention types–to move beyond structural co-occurrence and enable a multidimensional analysis of strategic partnership suitability.

This study focuses on clinical trials for stroke and diabetes, two conditions with significant global health impacts and overlapping risk factors ^5^. Both conditions share overlapping risk factors, such as hypertension, obesity, and sedentary lifestyles, making them relevant for studying collaborative clinical research networks ^6^. For instance, clinical trials for stroke and diabetes often involve multiple research centers across different geographical locations, each contributing unique datasets and expertise to the study. This complexity necessitates advanced modeling techniques to effectively map and optimize these collaborations ^7^. Analyzing these conditions allows us to explore how institutions collaborate in trials addressing common comorbidity and shared prevention strategies. Furthermore, the selection of stroke and diabetes demonstrated the versatility and applicability of our iGraphCTC model across different chronic diseases, illustrating its capability to handle diverse clinical trial data and provide valuable insights into collaborative networks.

Our proposed model significantly improves recommendation accuracy by considering user-user interactions and integrating supplementary clinical information. By incorporating additional clinical insights into the recommendation procedures, our approach elevates the precision of recommendations, presenting the limitations of mere user-user interactions commonly found in existing models. The novelty of iGraphCTC lies in its ability to incorporate supplementary clinical data into the recommendation process, offering a multidimensional analysis of affiliations and interactions. This represents a significant advancement over existing graph-based models, which primarily focus on surface-level user-user relationships without leveraging domain-specific insights into the data. Through the development and evaluation of iGraphCTC, this study contributes a robust framework for optimizing collaboration networks in clinical trials. By addressing the aforementioned research questions, we aim to provide actionable insights that support strategic decision-making for both researchers and sponsors in the field of chronic disease clinical research.

In other words, we aim to introduce a collaborative network framework that comprehensively considers collaborators and sponsors to optimize strategic partnerships in clinical trials. Our research questions (RQs) are as follows:

- **RQ 1**: What is the collaborative nature in clinical trials for chronic diseases?
- **RQ 2**: Is it valuable to identify the key affiliations for clinical trials of chronic disease through collaboration network analysis?
- **RQ 3**: How can the graph model be refined to optimize the efficiency of network recommendations in clinical trials?

Our RQs focus on improving and optimizing strategic collaboration in clinical trials. To effectively investigate the RQs, we gathered a dataset of clinical trials from ClinicalTrials.gov, one of the largest clinical trial databases worldwide ^8^. The collected data were processed using Python and used to build a graph model for collaboration network analysis. The remainder of this paper is organized as follows: Section Literature review presents an overview of prior research, and Section Study methodology explains the collected datasets. The recommendation modeling approaches and results are summarized in Sections Network construction and Results, respectively.

To ensure reproducibility and facilitate further research, we make our code and dataset publicly available on GitHub. The dataset used in this study was obtained from ClinicalTrials.gov, an open-access database, and is publicly available. All the necessary scripts, data, and instructions to replicate our analysis can be accessed at: https://github.com/dxlabskku/iGraphCTC.

## Literature review

### Network analysis of collaborative research

Most prior research on medical and clinical trials focused on individual research activities or gene/chemical networks^9–12^. However, considering that collaborative efforts among diverse institutes can enhance the efficiency and effectiveness of clinical and academic research^13–16^, several simultaneous collaboration network analyses were examined^17^.

^18^suggested that international collaborations in surgical practice can yield rapid and widely practical results, demonstrating that global strategies can overcome barriers to collaboration. They indicated the current state of clinical trials in surgical practice and proposed practical examples related to the direction of institutional collaboration.

Moreover,^19^ investigated the pattern and characteristics of collaboration networks among Iranian osteoporosis researchers through co-authorship social network analysis. They collected all articles published by Iranian researchers from 2009 to 2019 from three reputable academic databases: PubMed, SCOPUS, and Web of Science. After identifying Iranian researchers with more than five articles in the last ten years, they provided several academic guidelines and potential collaborators in the field of osteoporosis through an analysis of authorship graphs, co-authorship, and organizational/national networks.

^20^investigated the collaboration network of the Clinical and Translational Science Award, operated by the National Institute of Health (NIH) and the National Center for Advancing Translational Sciences. They proposed a link prediction model designed to identify potentially successful collaborations. By computing various network centrality measurements and considering the relative influences of each researcher, along with well-known prediction models (e.g., PageRank and Markov Model), they achieved per-network recommendations. Recently, several scholars directed their attention towards scale-free network analysis in the context of clinical trials^21^.

^22^developed a knowledge graph using unstructured clinical data primarily comprised of English narrative. While the system demonstrates proficiency in generating a biomedical knowledge graph from clinical data via the application of BERT models, it has a limitation in its concentrated focus on a specific phase of the data lifecycle, lacking thorough engagement with the analytical methods essential for a comprehensive exposition of the graph construction procedures.

We built subgraphs centered on disease-specific clinical trials, facilitating a better understanding of network dynamics within specific medical areas. This approach allows for the identification of influential collaborators and main institutions, providing a strategic roadmap for stakeholders seeking effective partnerships in clinical-trial research.

### Global collaboration and related recommendations in trials

The importance of international collaboration in surgical clinical trials has been emphasized, with organizations conducting clinical trials increasingly leveraging global networks to reduce costs and accelerate participant recruitment^23,24^. Most prior research contributed novel analyses and insights into identifying active groups involved in developing new indications and ingredients via global clinical trials. The main focus is on assisting institutes in finding information about global clinical trials. In contemporary practice, international collaboration is facilitated through agreements among organizations, universities, hospitals, and companies, mainly leading to the formation of research groups^25^. Active research and trials can be conducted more efficiently by leveraging networks with groups already engaged in research, thereby reducing operational costs, promptly recruiting a large number of patients, and broadening the geographic dispersion of drug development operations^26^. Drawing on a literature review of prior research, which highlighted the advantages of clinical cooperation networks and open innovation, this study aims to conduct further analysis and provide assistance in identifying such collaboration networks.

^27^investigated the distribution of clinical trials and found that while a significant number of trials are conducted in the United States, there is also a substantial proportion of trials being conducted internationally. With the growing importance of global participation in clinical trials, it is necessary to consider ethnic and international factors that affect the results, particularly in areas such as Alzheimer’s disease (AD). Using our clinical trial dataset for Diabetes and Stroke, we observed similar trends, with a notable number of trials conducted in the United States and a substantial proportion internationally. This comparison highlights the importance of international collaboration in chronic disease research and underscores the need to optimize these collaborative networks, as proposed in the iGraphCTC model.

Therefore, the purpose of the current study is to provide novel analyses and insights into the global network of international clinical trials. This can be achieved by identifying active groups within the clinical trial network, investigating groups that are specifically focused on the development of new indications and components, and recommending affiliations with extensive experience in group collaboration and similar trials. The main intention is to furnish institutions facing challenges in accessing detailed information on the global clinical trial network and provide guidance for establishing collaboration networks.

Based on a literature review, this study re-examines the advantages of clinical collaboration networks and the significance of open innovation. The anticipated outcome is practically valuable for stakeholders in identifying networks for potential collaboration, particularly through the application of the proposed graph-based recommendation model, iGraphCTC. It is primarily designed to identify and connect researchers with potential collaborators and sponsors that align with their specific disease areas. By facilitating these strategic collaborations, iGraphCTC indirectly supports the design and execution of clinical trials, ensuring that trials are well-supported and effectively managed.

## Study methodology

### Data description

We collected clinical trial datasets from the ClinicalTrials.gov database^28^, specifically on chronic diseases, including 1) ‘diabetes and 2) ‘stroke’ or ‘Cerebrovascular Accident (CVA)‘, mentioned in either the ‘Study title’ or ‘Conditions’ fields. The list of clinical trials was directly provided in a spreadsheet format, while the exported search results were presented in a CSV format with seven fields: ‘NCT Number’,’ ‘Study Title’,’ ‘Conditions’, ‘Interventions’,’ ‘Sponsor’, ‘Collaborators’, and ‘Locations’. Each of the seven fields in the CSV format plays a critical role in our analysis.

- **NCT Number**: A unique code assigned to each clinical trial, allowing for easy tracking and reference across different databases and studies.
- **Study Title**: A brief summary of the trial’s purpose and focus, aiding in the classification and understanding of its objectives.
- **Conditions**: Specifies the medical conditions targeted by the trial, essential for categorizing and analyzing disease-specific collaborations and research trends.
- **Interventions**: Details the treatments or procedures being tested.
- **Sponsor**: Identifies the organization funding and overseeing the trial, critical for analyzing funding sources, institutional collaborations, and potential conflicts of interest.
- **Collaborators**: Lists other organizations or institutions participating in the trial, necessary for mapping collaborative networks and understanding the distribution of research efforts.
- **Locations**: Indicates where the trial is being conducted, important for geographical analysis, understanding regional research strengths, and identifying potential areas for expanding collaborations.

We created two columns within our dataset: ‘Affiliations’, including both collaborators and sponsors, and ‘﻿Interventions’, categorizing the type of clinical trials (e.g. DRUG, BEHAVIORAL, DEVICE, and others). Subsequently, data that were not directly related to each disease were reviewed and excluded to ensure a focused and relevant dataset for analysis.

### Preprocessing

To facilitate the training and testing of the graph models, the data underwent a specific transformation process. The data in the datasets were extracted and standardized using Python Pandas. The names of collaborators, sponsors, and locations in each study were separated using the delimiter ” ‘|’. Country names were extracted into the ‘﻿Location’ column. Each row contains information about an individual clinical trial.

To examine the data for assessing research collaborations, the abbreviations of affiliations were unified, and the dataset was filtered to select only the affiliations. Additionally, hospital selection is crucial in clinical trials, and in many countries, hospitals are often affiliated with universities. In such cases, the hospital was renamed using the university name^29^. Owing to variations in presenting the same affiliation (e.g., Harvard University Medical School/Harvard Medical School), all affiliations were manually reviewed and cross-checked in the datasets. Furthermore, non-English affiliations were revised to reflect their English equivalents. Using these procedures, this study identified 2,353 affiliations in 17,259 clinical trials related to diabetes and 4,579 affiliations in 50,018 clinical trials related to stroke.

### Societal benefits and key determinant

This study aims to enhance the efficiency and reach of clinical trials for chronic diseases, such as diabetes and stroke, by leveraging robust network analyses. In practical terms, making trials faster and more cost-effective brings substantial social benefits; it enables the earlier identification of effective treatments, lessens the financial strain on healthcare systems, and accelerates the delivery of innovative therapies to those in need. Consequently, the key factor driving societal impact is effective collaboration among diverse stakeholders. When research institutions, hospitals, pharmaceutical companies, and governments pool their resources and expertise, the likelihood of successfully conducting large-scale clinical trials in a timely manner markedly increases.

By offering a graph-based framework for optimizing and recommending collaborative ties, iGraphCTC minimizes resource duplication and operational costs. This improvement is especially critical for under-resourced regions that face challenges in joining global clinical trials. Through strategic partnerships, these regions can broaden patient recruitment, diversify participant pools, and ensure that new treatments are tested in various demographic settings. In essence, the iGraphCTC’s ability to identify and recommend high-value partnerships is key to enhancing both equity and innovation in clinical research.

## Network construction

### Graph generation

**Fig. 1:**
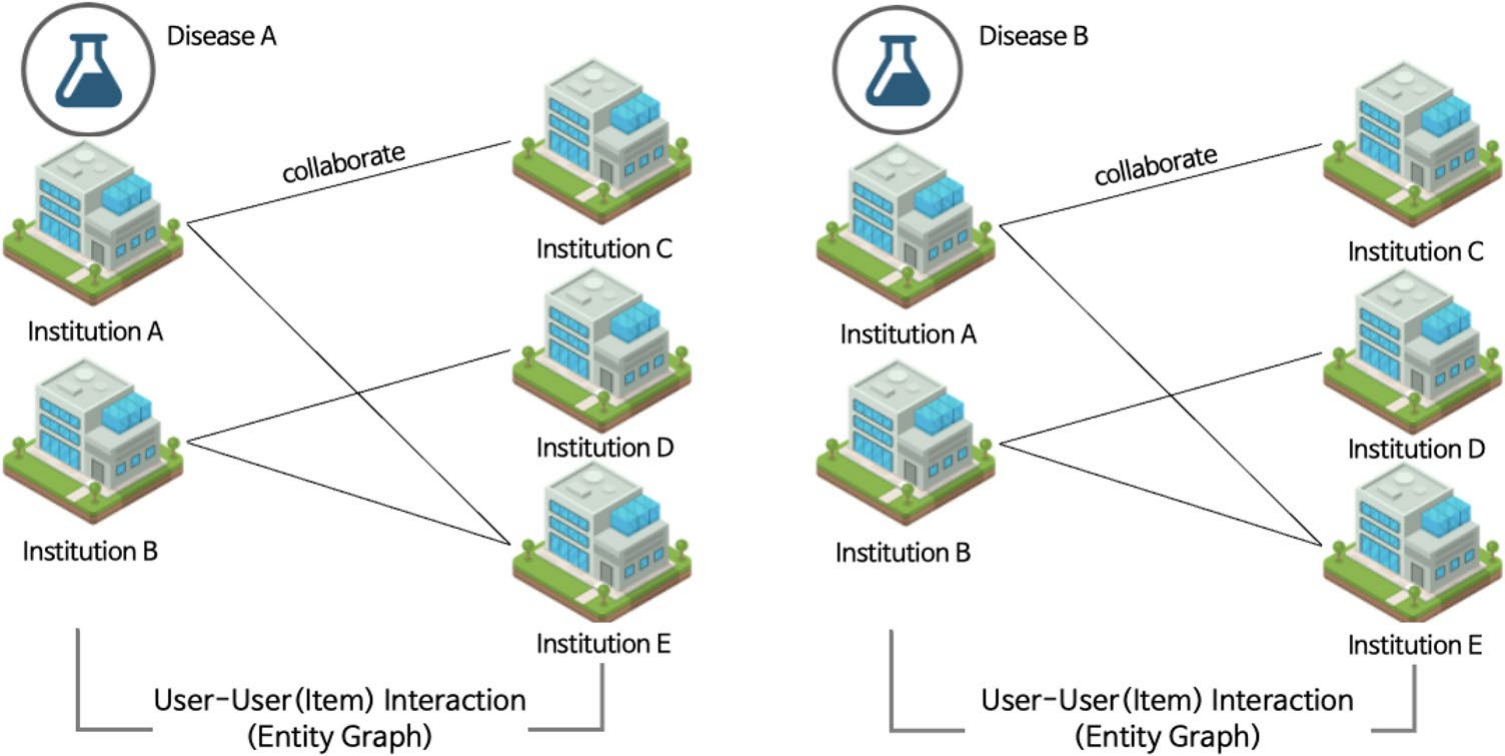
Example of the research collaboration network model.

The affiliations within the dataset are linked to institutions that have conducted the same clinical trials. The research collaboration network comprises these affiliations. Using the available clinical datasets of affiliations, a network matrix representing collaboration between research institutions was constructed. This study introduces a cumulative weighted graph network, an undirected collaboration network, to observe the dynamics of a clinical trial collaboration network. Figure 1 illustrates an example of the collaboration network used in this study. A single institution may collaborate with several research institutions, and each affiliation can form numerous partnerships with other affiliations. To capture the depth of these collaborations, a cumulative weighted graph network was employed, acknowledging that affiliations may engage in multiple collaborative efforts. This network is generated using a Dynamic Graph Library (DGL)^30^. The DGL library in Python aids in comprehending the collaboration network of research institutions, providing the capability to employ computational network modeling and implement a highly flexible graph^31^.

We constructed a DGLGraph by representing our matrix of affiliations as an adjacency list. Each node uniquely identified an institution, and edges were established based on shared participation in clinical trials. We then integrate this information into the graph structure by adding nodes and edges. Subsequently, we employed DGL’s built-in neural network modules (dgl.nn) to implement and train the selected GNN architectures (GCN, GraphSAGE, and GAT) on the constructed graph. This approach allowed us to handle multi-attribute data, streamline the embedding process, and efficiently perform link prediction tasks across different subgraphs.

The graph properties used in this study are defined as follows:

- **Node:** Each node represents a distinct affiliation. The total number of nodes (*N*) corresponds to the total number of affiliations in the dataset.
- **Edge:** An edge symbolizes the connection between two affiliations, which collaborated on a single clinical trial.
- **Node Weight:** Each node weight signifies the centrality of an affiliation, quantified by the total number of clinical trials in which the affiliation participated. This measure highlights the overall activity of a node within the network. The node weight is not the sum of the edge weights but represents the node’s participation in clinical trials. This finding highlights the most influential research affiliations in clinical trials for each disease type.
- **Edge Weight:** Each edge weight corresponds to the number of connections between two affiliations, reflecting the strength and frequency of their direct interactions in clinical trials. A higher edge weight indicates a greater number of collaborations between the two affiliations in clinical trials. Both node and edge weights are used to compute node embeddings, capturing different dimensions of the graph. Node weight provides a measure of centrality that highlights the overall activity level of each affiliation in the clinical trials. Edge weights emphasize the strength of collaborative relationships and ensure a comprehensive representation of the networks.

### Baseline models

As our baselines, we benchmarked iGraphCTC against two machine learning approaches and seven established GNN architectures: XGBoost, logistic regression, GCN, GraphSAGE, GAT, iMovieRec, GNN-IR, and DHGT-DTI. Because our primary contribution is centered on data adaptation and feature integration rather than algorithmic innovation, these comparison models serve as essential control groups. They represent the performance ceiling for standard GNN applications in this domain, which mainly rely on the underlying graph structure. The comparison unequivocally demonstrates that iGraphCTC’s superiority is attributable to the integration of clinical domain-specific attributes (geographical and intervention data), validating our framework’s approach.

- **Graph convolutional network (GCN):** GCN is a type of neural network designed to directly operate on graphs, utilizing their structural information^32^. GCN serves as a foundational model in the GNN domain, demonstrating the effectiveness of spectral graph convolutions for node representation learning.
- **Graph Sample and AggregatE (GraphSAGE):** GraphSAGE is a framework designed for inductive representation learning on large graphs. Unlike a GCN, which is transductive and works with a fixed graph, GraphSAGE learns a function to generate embeddings by sampling and aggregating features from a node’s local neighborhood^33^.
- **Graph Attention Network (GAT):** GAT introduces the attention mechanism into the domain of graph neural networks. It assigns different weights to different nodes in a neighborhood, enabling attention allocation to nodes when aggregating information^34^.
- **iMovieRec:** iMovieRec is a hybrid recommendation method utilizing graph features by efficiently learning the interactions between users and items with single-layer neural networks and matrix factorization^35^.
- **GNN-IR:** GNN-IR, a graph neural network for influencer recommendation, based on the connections between companies and influencers of social media. It employs a data-driven methodology utilizing a meticulously curated dataset collected in-house by addressing diverse data modalities^36^.
- **DHGT-DTI:** It is a deep learning-based framework for addressing drug-target interactions prediction. It captures the local and global structural information of the network from both neighborhood and meta-path perspectives^37^.

### Proposed model

iGraphCTC presents an innovative approach for building an all-encompassing graph based on the clinical trial data. It functions as a graph-based recommendation system that discerns and forecasts potential collaborations in the domain of chronic disease clinical trials. Moreover, the model commences by dividing the overarching graph into disease-centric subgraphs. This division is significant because it allows for the incorporation of disease-specific dynamics into the network. The model harnesses geographical and intervention data as multifaceted attributes, improving the graph with layers of contextual information beyond the fundamental clinical trial connections.

The iGraphCTC model is an adapted GCN framework built for the specific task of clinical trial collaboration recommendation. The core conceptual advancement is the inter-connected feature fusion architecture. This architecture is designed to incorporate supplementary clinical data by transforming high-dimensional attributes into semantic embeddings that are fed directly into the GCN layers. Thus, iGraphCTC is a framework for multidimensional analysis, enabling recommendations based on both collaboration history and the semantic suitability derived from clinical attributes.

**Fig. 2:**
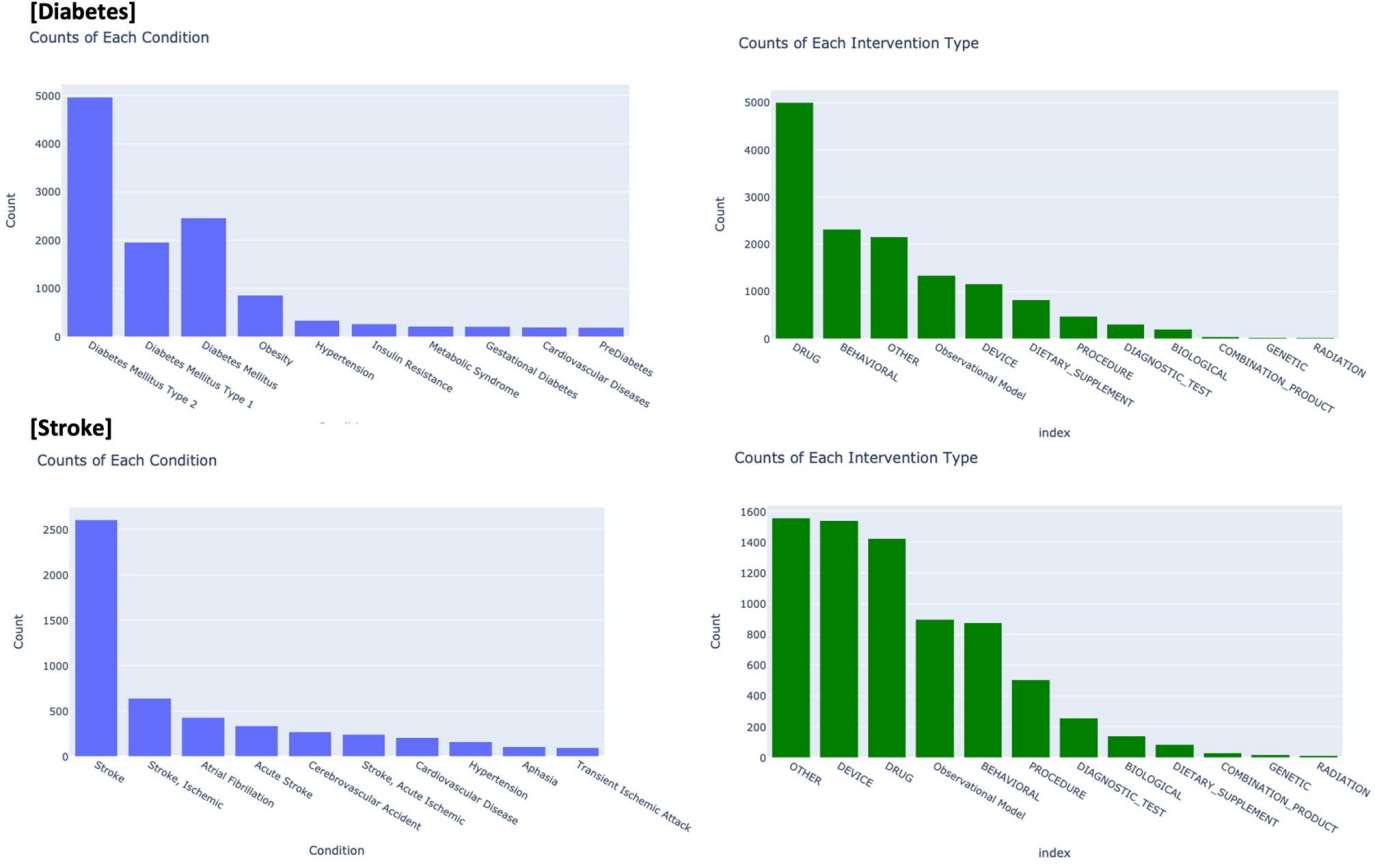
Statistics of extra attributes in Diabetes and Stroke trials. This provides an overview of the distribution of various conditions within the clinical trials dataset.

**Fig. 3:**
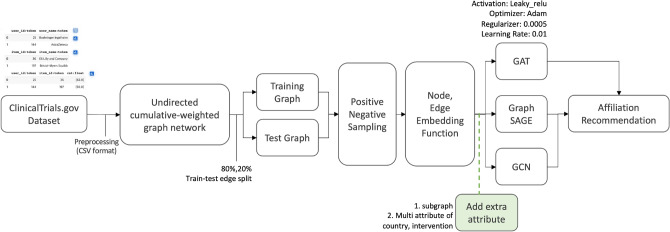
Overall recommendation procedures.

The distributions of the additional information in the trials are presented in Fig. 2. The isolated nodes were designated as the training set, whereas the remaining trials were randomly divided, with 80% allocated to the training set and the remaining 20% allocated to the test set. The overall modeling procedure for the iGraphCTC is shown in Fig. 3. The following sections present these details in order.

#### General construction

Although GNNs examined promise in general recommendation tasks, their application to specialized scientific domains like clinical trial collaboration often faces the critical challenge of data sparsity. Most existing GNN models predominantly learn representations based only on the graph structure (co-occurrence data), which is insufficient for new or less-active collaborations. In contrast, the conceptual novelty of our iGraphCTC model lies in its inter-connected architecture (Fig. 3. This architecture proactively incorporates rich external domain knowledge–specifically semantic embeddings of intervention types and geographic context–to augment the initial node features. This feature fusion strategy is essential for mitigating the sparsity issue, allowing the model to make informed recommendations based on both collaboration history and semantic suitability, a key distinction from previous graph-based collaboration systems.

The proposed iGraphCTC model is designed to overcome the limitations of traditional models by integrating multi-dimensional domain knowledge into the graph learning process. iGraphCTC enhances the node representation by fusing two critical clinical trial attributes: geographical location and normalized intervention types. This process moves beyond simple feature concatenation by creating an inter-connected representation where node features explicitly encode the semantic and contextual relevance of each affiliation (e.g., similar research focus or advantageous location) using high-quality semantic embeddings. This architectural choice is the central advancement, enabling the subsequent GCN layers to learn highly sophisticated, domain-aware embeddings that drive recommendations based on both past structural collaboration and future strategic suitability.

Our architecture incorporates geographical and design details from each trial, enhancing the applicability of the network by providing a multidimensional perspective of collaboration. Node attributes include the number of clinical trials (cnt) and the geographical location of the institution (country), introducing a spatial dimension to the graph. Edge attributes capture both the frequency of collaborations and a normalized measure of interventions, scaling the raw counts to a range of 0–1. This normalization ensures consistency and comparability across different edges, thereby facilitating effective incorporation into the GNN. This approach provides insights into the depth and nature of collaborative efforts between institutions. The comprehensive construction and analysis of the graph, supported by the robust capabilities of DGL and the advanced embedding techniques of OpenAI, establish a foundation for a transformative recommendation model in the field of chronic disease clinical trials.

**Fig. 4:**
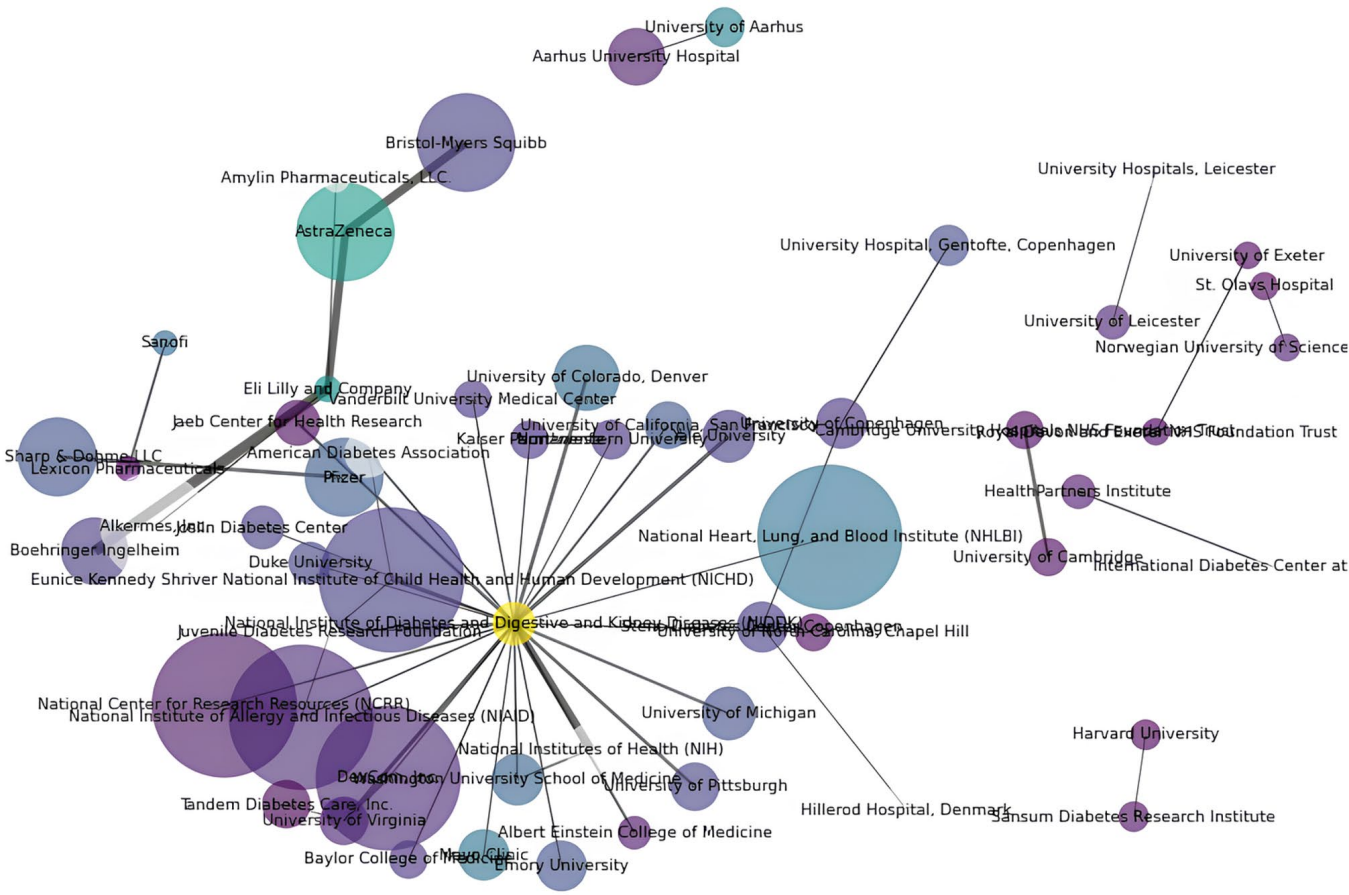
Sub-graph generated of diabetes clinical trials.

Although conventional GNNs mainly address structural co-presence, such approaches are likely to be insufficient for clinical trial collaboration networks because of data sparsity^38^. Compared with general feature concatenations, the main contribution of iGraphCTC is rooted in its domain-specific feature fusion approach. We strategically select intervention attributes not only as data, but also as proxies for institutional capability and physical accessibility, which are important factors in determining clinical trial success. By transforming these high-dimensional clinical contexts into semantic embeddings via the inter-connected architecture (Fig. 4), the model learns sophisticated, domain-specific latent representations that general-purpose GNNs cannot capture. This architectural design specifically addresses the cold-start problem of the domain, where the past collaboration history is absent.

#### Node feature processing

The extraction process begins with parsing the clinical trial data to identify the affiliations and their corresponding trials. Each affiliation is mapped to its respective intervention type and geographical location, forming the initial categorical data points for embedding. To convert these categorical attributes into dense vectors, we used the OpenAI model text-embedding-ada-002. This ensures the capture of semantic associations and improves the representation of the data in a vectorized shape^39^.

#### Recommendation generation

The core of the recommendation system is a collaborative filtering mechanism that scores potential affiliations based on their similarity and the strength of existing collaborations. Depending on the characteristics of graph neural networks (GNNs), iGraphCTC can predict the likelihood of future collaborations. It facilitates the establishment of strategic partnerships, accelerates clinical trial procedures, and enhances innovation in the treatment and management of chronic diseases. The recommendations of iGraphCTC are as follows:

The recommendation process transforms the final high-quality node representations learned by the *iGraphCTC* framework into a quantifiable score that predicts the likelihood of a collaboration between any two affiliations. This process, known as **Link Prediction**, is mathematically executed using a *Dot Product Predictor* ($\phi$).

### Step 1: GNN output (The learned embeddings)

After the input features (which include the fused attribute data) have propagated through the *L* layers of the Graph Convolutional Network (GCN), the model generates a final, *d*-dimensional embedding vector for every node. These vectors are the ultimate output of the *iGraphCTC* framework, encoding the affiliation’s position in the network, their collaboration history, and their strategic suitability based on the integrated attributes.

The final embeddings for two affiliations, *u* and *i*, are defined as:

$$\textbf{h}_u = \text {iGraphCTC}(\textbf{X}_u, \textbf{A}) \quad \text {and} \quad \textbf{h}_i = \text {iGraphCTC}(\textbf{X}_i, \textbf{A})$$

where:

- $\textbf{h}_u$ and $\textbf{h}_i$ are the final *d*-dimensional embedding vectors for Affiliations *u* and *i*, respectively. These vectors encode the comprehensive, learned representation.
- $\textbf{X}$ is the comprehensive node feature matrix (with fused attributes).
- $\textbf{A}$ is the graph’s adjacency matrix.

### Step 2: Collaboration score calculation

The collaboration score ($s_{ui}$) between two potential partners is calculated by measuring the proximity or similarity between their final embeddings ($\textbf{h}_u$ and $\textbf{h}_i$) in the learned latent space. The Dot Product function efficiently measures this proximity. The core hypothesis is that affiliations that are strategically suited for collaboration will have similar embeddings.

The collaboration score is mathematically expressed as:

$$s_{ui} = \phi (\textbf{h}_u, \textbf{h}_i) = \textbf{h}_u^\top \textbf{h}_i$$

The output $s_{ui}$ is a single scalar value that represents the unnormalized probability of a collaboration link existing between *u* and *i*.

### Step 3: Recommendation ranking

The system calculates the collaboration score for all potential non-existent edges (i.e., potential partnerships) in the network. These scores ($s_{ui}$) are then ranked in descending order, and the affiliations corresponding to the top *N* highest scores are recommended to the user as the most strategically suitable partners for future collaboration.

As detailed, the following explanations are presented.

1. Node embedding and edge definition
  $G = (V, E)$ represent the original graph, where $V$ represents the set of nodes (affiliations) and $E$ represents the set of edges (collaborative relationships).
  Each node $v_i \in V$ has an associated embedding $h_i$ derived from the node embedding process using the OpenAI text-embedding-ada-002 model. This model processes the textual information associated with each node into dense vector representations, thereby capturing the characteristics of the affiliation.
2. Positive and negative edge constructions for recommendations
  For a target node $v_u$, define ${E}_u$ as the set of its current collaborative relationships (positive edges).
  Construct a set $E_u^-$ of negative edges for $v_u$ where $E_u^- = \{ (v_u, v_i) | v_i \in V, v_i \notin {E}_u \}$.
  The comprehensive graph for recommendations, $G_u = (V, E_u \cup E_u^-)$, includes both existing and potential collaboration for $v_u$.
3. Collaboration score computation
  Employ a scoring function, such as a DotPredictor, denoted as $\phi$, to calculate the collaboration score for each edge in $E_u \cup E_u^-$.
  The collaboration score for an edge $(v_u, v_i)$ is given by $s_{ui} = \phi (h_u, h_i)$.
  A higher dot product implies more similarity (or stronger alignment) between embeddings, reflecting a higher potential for meaningful collaboration. The DotPredictor score directly determines the ranking of edges in the recommendation system. Affiliations whose embeddings produce higher dot products with a given target node are more likely to form beneficial partnerships. This scoring mechanism underpins the model’s ability to prioritize or filter out potential edges in an interpretable, scalable manner.
4. Ranking and recommendation
  Rank the potential collaborations based on their scores: $S_u = \{ (v_i, s_{ui}) | (v_u, v_i) \in E_u \cup E_u^- \}$, sorted by $s_{ui}$ in descending order.
  Recommend the top affiliations based on the ranked list $S_u$, considering both existing and new collaboration potentials.

For an affiliation $v_0$ (*user_id*=0), an implementation example of a recommendation is as follows:

1. Identifies $F_0$, the set of current collaborations (positive edges).
2. Constructs $G_0$ with both positive and negative edges $E_0 \cup E_0^-$.
3. Calculates scores $\{ s_{0i} \}$ for each potential collaboration in $G_0$.
4. Ranks these scores to suggest the top N affiliations as potential collaborators, considering both established and potential partners.

#### Evaluation metrics

In iGraphCTC, we employ three key metrics to evaluate the performance of our model: Area Under the Curve (AUC), F1-score, and accuracyK^40–42^. This metric measures the proportion of recommended items in the top*k* list that are relevant to the user’s affiliation.

- **Area Under the Curve (AUC)** is calculated as :
  1 $$\begin{aligned} \int _{0}^{1} \frac{\text {True Positive Rate (TPR)}}{\text {False Positive Rate (FPR)}} \, d(FPR) \end{aligned}$$
- **F1-score** is calculated as:
  2 $$\begin{aligned} 2*\frac{Precision*Recall}{Precision+Recall} \end{aligned}$$
- **Accuracy@K** is calculated as :
  3 $$\begin{aligned} \frac{1}{N} \sum _{i=1}^{N} {| \text {Rel}_i \cap \text {Rec}_i(k) |} \end{aligned}$$

where *N* is the number of users, $\text {Rel}_i$ is the set of items relevant to user *i*, and $\text {Rec}_i(k)$ is the set of top-*k* items recommended to user *i*.

### Experimental setup

To ensure the validity and practical relevance of our model, the experiments were designed around a link prediction task, where the objective is to predict future collaborative partnerships (edges) between existing affiliations (nodes). First, to simulate a realistic scenario of predicting future collaborations, we implemented a temporal data split. The collaboration data (edges) derived from clinical trials registered between January 1, 2011, and December 31, 2021, were allocated to the training set. The most recent collaborations registered in the subsequent year, 2022, were exclusively reserved for the test set. This methodology ensures that the model is evaluated on its ability to generalize to unseen, future collaborations. Furthermore, to ensure the statistical reliability of our results and minimize potential bias from random weight initialization and the selection of negative samples, the entire training and evaluation procedure was repeated 10 independent times. Second, the training and testing of a link prediction model require the generation of negative samples (non-existent or false edges). For the training set, negative edges were generated by randomly sampling non-connected node pairs in the graph, ensuring a 1:1 ratio with positive (existing) edges. For the test set, the negative edges were generated by pairing test-set nodes with random non-collaborating nodes, ensuring they also did not appear in the training positive set. This rigorous negative sampling ensures a balanced and challenging evaluation. Third, all GNN models (iGraphCTC and baselines) were implemented using the PyTorch and Deep Graph Library (DGL) frameworks. Key hyperparameters were uniformly set after preliminary tuning: the learning rate was set to 0.001, the embedding dimension for node features was set to 128, and the number of GCN layers was set to 2. We utilized the Adam optimizer with L2 regularization ($10^{-5}$) and trained for 100 epochs with early stopping based on validation loss^43–45^.

To make sure the integrity and robustness of the evaluation and prevent indirect data leakage, we strictly separated the attribute extraction procedures from the target labels (collaboration links). In particular, geographical and intervention attributes are extracted solely from the metadata of individual clinical trials available at the time of the trial’s registration, independent of any future collaboration outcomes. Furthermore, all attributes used in the training phase were strictly confined to the training split, ensuring that the model did not utilize any look-ahead information from the test set.

## Results

### Network analysis

To address RQ1, “*What is the collaborative nature in clinical trials for chronic diseases?*”, We began by constructing cumulative weighted undirected graphs for diabetes and stroke. Nodes represent distinct affiliations (universities, research institutes, hospitals, or pharmaceutical companies), and edges indicate shared participation in one or more clinical trials. It is intuitive that nodes located centrally within clusters have a higher number of collaborations owing to the graph design. This observation validates the effectiveness of the iGraphCTC model. By accurately capturing these expected patterns, the model demonstrates its reliability in reflecting real-world collaborative dynamics in the network.

Figure 4 displays a diverse range of affiliations that exhibit varied collaboration patterns across different locations and conditions. The figure illustrates these collaboration dynamics using node colors to represent the number of unique conditions, node sizes to indicate the number of collaborations, and edge thickness to show the frequency of collaborations. Detailed legends and color scales are included in the figure for clarity purposes.

The visualization reveals two key patterns in chronic disease clinical trials: (1) Clustered Collaboration, affiliations with common research interests form dense sub-communities within the larger network; and (2) Central Influencers, a small number of nodes display notably high centrality, often due to their repeated participation in large-scale multinational trials. These nodes are potential hubs that connect collaborators, highlighting the importance of strategically aligning them for efficient knowledge transfer and resource sharing.

### Model performance

We need to present the model performance by using a suite of evaluation metrics to affirm its predictive accuracy and generalizability. The validation is required to be conducted for a rigorous comparison with baseline models and an analysis of the precision and recall rates of the recommendation system. In order to address RQ2, “*Is it valuable to identify key affiliations for clinical trials of chronic disease through collaboration network analysis?*”, and RQ3, “*How can the graph model be refined to optimize the efficiency of network recommendations in clinical trials?*”, we compared the proposed iGraphCTC framework with three established graph-based models: GCN, GraphSAGE, and GAT. This comparison involved a comprehensive set of evaluation metrics, including AUC, F1-score, and accuracy@K, to ensure a rigorous assessment of predictive accuracy and overall model generalizability.

Our iGraphCTC model, which was enhanced with multi-attribute considerations, demonstrated superior performance across various metrics. It outperformed the baseline models in recommending viable collaborators, as evidenced by higher accuracy and F1-score, especially when the model utilized multiple attributes, such as geographical and intervention data.

Furthermore, we examined the performance boost provided by the iGraphCTC model using multiple strategies. This included adding significant attributes by creating subgraphs and incorporating multiple attributes within the graph, which contributed to the improved accuracy and effectiveness of our model.

Table 1 summarizes the performance of various models in recommending affiliations for diabetes clinical trials using metrics like AUC, F1-score, and accuracy@K (top-K recommendations) and Table 2 provides performance metrics for recommending affiliations in stroke-related clinical trials. These results further validate the robustness of iGraphCTC across different diseases. Our proposed iGraphCTC model, which incorporates multiple attributes through the GCN framework, outperformed the other models for both diabetes and stroke. It demonstrated an AUC of 88.18% and 79.59%, an F1-score of 84.16% and 69.57%, and an accuracy range of 8.29-24.57% and 3.19-11.18% across various accuracyK metrics. These substantial improvements underscore the effectiveness of the proposed model.

**Table 1:** Performance evaluations (%) for diabetes clinical trials.

Model	AUC	F1-score	acc@1	acc@2	acc@3	acc@4	acc@5	acc@6	acc@7	acc@8	acc@9	acc@10
XGBoost	30.44	31.45	0.01	0.81	0.91	0.98	1.03	1.24	1.35	1.35	1.37	1.40
Decision tree	29.42	30.97	0.05	0.87	0.97	0.97	1.00	1.10	1.15	1.17	1.26	1.34
GCN	71.47	72.09	4.09	4.81	6.01	6.49	7.93	8.65	8.89	8.89	9.86	10.10
GraphSAGE	70.85	68.65	0.72	1.44	2.16	3.13	3.61	4.81	5.29	6.01	6.49	6.73
GAT	73.99	68.80	0.00	0.24	0.48	0.72	0.72	1.20	1.44	1.92	3.61	4.57
iMovieRec	72.23	69.80	2.12	2.54	4.15	4.27	4.85	6.75	6.30	7.98	7.96	8.64
GNN-IR	74.40	72.20	4.25	6.52	7.11	6.33	6.37	9.13	8.02	11.52	8.98	11.84
DHGT-DTI	76.88	74.14	5.73	8.20	9.18	9.76	7.85	13.78	11.54	13.41	13.77	14.41
iGraphCTC (Subgraph)	70.24	60.87	3.83	4.92	6.01	8.20	8.74	9.29	10.38	13.11	13.66	13.66
iGraphCTC (Multi-attribute w/ GCN)	**88.18**	**84.16**	**8.29**	**11.71**	**14.00**	**16.00**	**17.14**	**18.57**	**20.29**	**22.86**	**23.71**	**24.57**
iGraphCTC (Multi-attribute w/ GraphSAGE)	87.15	83.58	8.00	10.86	13.71	14.29	15.71	18.00	19.14	19.14	20.00	21.14

**Table 2:** Performance evaluations (%) for stroke clinical trials.

Model	AUC	F1-score	acc@1	acc@2	acc@3	acc@4	acc@5	acc@6	acc@7	acc@8	acc@9	acc@10
XGBoost	27.11	14.37	0.01	0.50	0.54	0.55	0.70	0.71	0.74	0.75	0.80	0.81
Decision tree	27.19	17.21	0.04	0.06	0.14	0.20	0.74	0.75	0.79	1.01	1.02	1.03
GCN	69.47	49.42	0.00	1.90	3.81	4.29	6.67	6.67	6.67	6.67	7.62	8.10
GraphSAGE	65.81	49.02	0.48	0.95	1.90	2.38	3.81	3.81	3.81	4.29	4.29	4.76
GAT	69.07	59.36	0.00	0.00	0.00	0.00	0.00	1.35	1.35	1.35	1.35	1.35
iMovieRec	67.32	50.02	1.71	2.01	3.51	3.44	4.88	5.55	5.03	5.70	6.15	6.05
GNN-IR	70.86	53.79	2.96	3.58	5.03	6.66	7.19	8.16	8.17	8.44	7.33	7.85
DHGT-DTI	72.46	55.16	2.25	3.37	5.81	6.52	7.72	8.12	8.26	8.39	9.42	9.84
iGraphCTC (Subgraph)	59.63	57.26	0.00	1.10	1.65	2.75	4.95	5.49	6.04	6.04	6.04	6.59
iGraphCTC (Multi-attribute w/ GCN)	79.59	69.57	**3.19**	**4.15**	**6.39**	**7.99**	**8.63**	**8.95**	**8.95**	**9.90**	**10.22**	**11.18**
iGraphCTC (Multi-attribute w/ GraphSAGE)	**81.01**	**71.00**	0.32	2.56	4.79	5.43	6.07	7.03	7.03	7.99	8.95	9.58

### Practical utility and strategic implications

Based on the results, we can translate them into actionable strategic intelligence for clinical trial sponsors. The model’s performance has direct implications for reducing costs, accelerating trial timelines, and optimizing resource allocation.

A key benefit is demonstrated by the model’s performance on *Accuracy*@*K*. If a sponsor typically evaluates $K=10$ potential collaborators, iGraphCTC’s superior accuracy (e.g., $17.44\%$ higher than baselines in some subsets) means a significantly higher probability that a truly viable partner is found within that initial short-list. This translates directly to reduced administrative overhead and a shorter cycle time for collaborator selection. Moreover, the high F1-score confirms that the model achieves an optimal balance between precision (avoiding irrelevant partners) and recall (not missing highly suitable partners). In a real-world scenario, this mitigates two major risks: wasting resources by pursuing unsuitable leads (false positives) and missing a strategically perfect collaborator (false negatives).

We also address a pharmaceutical sponsor seeking a Phase III trial partner for a novel diabetes intervention (a new drug class). A traditional GCN, relying solely on collaboration history, might recommend a large academic center that has participated in many diabetes trials but is geographically distant and has focused primarily on older insulin therapies. The recommendation is structurally ’likely’ but semantically weak. Our model, iGraphCTC, efficiently utilizes the inter-connected feature fusion, and learns the semantic suitability of the intervention type. It is trained to recognize that the new drug class requires expertise in specific metabolic pathways. The model correctly elevates a smaller, more specialized research institute that has recently co-authored papers on the specific metabolic pathway, despite having fewer overall historical collaborations, and is located in an advantageous logistical zone.

The practical utility of iGraphCTC is that it transforms the recommendation task. It moves beyond identifying collaborators who might work together (based on history) toward identifying those who are strategically optimal for the precise clinical and logistical requirements of the current trial. The model’s high predictive accuracy serves as a powerful validation that its feature integration framework successfully captures the complex, multidimensional nature of effective clinical collaboration.

## Discussion and conclusion

This study introduces iGraphCTC and explores its potential for utilizing graph-based methodologies to recommend affiliations, such as universities, research institutes, hospitals, and pharmaceutical companies, for clinical trials. Using the ClinicalTrials.gov dataset, a graph was constructed in which distinct affiliations were depicted as nodes and edges quantified collaborative efforts based on the aggregate number of jointly conducted clinical trials.

iGraphCTC, which employs an innovative graph-based recommendation system, exhibited superior predictive performance in identifying viable collaboration partners compared with established models such as GCN, GraphSAGE, and GAT. The primary goal of iGraphCTC is to optimize collaboration networks by connecting researchers with suitable collaborators and sponsors, ultimately enhancing the design and execution of clinical trials. This application-centric approach is a key aspect of our work, addressing the unique challenges of forming strategic collaborations in clinical trials for chronic diseases.

To address the primary research question presented in Fig. 4, clinical trials exhibit intricate collaborations among diverse stakeholders, including pharmaceutical companies, research institutions, universities, and hospitals. By visualizing these collaborations, Fig. 4 identifies key players, understands collaboration patterns, allocates resources effectively, and facilitates strategic decision-making. This, in turn, enhances the formation and optimization of partnerships. These collaborations are pivotal for consolidating resources, expertise, and data, thereby enabling more comprehensive and diverse clinical trial designs. Furthermore, this study underscores the significance of network analysis in understanding collaborative dynamics through a comprehensive review of network collaboration in clinical trials.

As presented in the Network Construction section, leveraging graphs to identify key affiliations aids strategic decision-making and enhances the efficiency of clinical trials. Finally, Tables 1 and 2 present the iGraphCTC model’s proposition, which incorporates multifaceted attributes that significantly bolster recommendations. This study advances the understanding of chronic disease clinical trials by demonstrating the effectiveness of network analyses in depicting collaboration dynamics and identifying potential collaborators.

Regarding the first RQ, as highlighted in Fig. 4, clinical trials exhibit intricate collaborations involving a spectrum of stakeholders, such as pharmaceutical companies, research institutions, universities, and hospitals. These partnerships play a pivotal role in consolidating resources, expertise, and data, thereby facilitating comprehensive and diverse clinical trial design. Moreover, our literature review emphasizes the significance of network collaboration in clinical trials. Network analysis is a valuable tool for unraveling the intricacies of these collaborations. As evidenced in the Network Construction section, leveraging graphs to identify key affiliations is instrumental in strategic decision-making, ultimately enhancing the efficiency of clinical trials.

Moreover, as shown in Tables 1 and 2, we introduce the iGraphCTC model, which significantly improves recommendation accuracy by incorporating multifaceted attributes. This study contributes to advancing the comprehension of clinical trials in chronic disease research, which is a crucial and pertinent medical domain. This demonstrates the efficacy of network analyses in delineating collaborative activities within these domains and identifying potential collaborators.

Beyond merely identifying high-impact institutions, the model also has the capacity to predict emerging collaborative opportunities. By systematically flagging partnerships that are likely to yield mutual benefits, iGraphCTC offers a strategic roadmap for sponsors, research institutions, and policymakers to optimize their resource allocation. Overall, these findings underscore the practical value and scalability of iGraphCTC as a powerful recommendation system for chronic disease research.

However, the study faced certain limitations that warrant acknowledgment. First, future studies should explore integrating dynamic data sources and expanding the model’s applicability to diverse disease areas which could further enhance its predictive accuracy and adaptability. Exploring real-time data integration can augment the model’s predictive capabilities and broaden its adaptability. Second, the manual standardization of affiliation names, while necessary for data integrity, posed challenges in scalability and introduced potential human bias. Automating the data cleaning and standardization process using natural language processing (NLP) techniques can also improve scalability and minimize bias, ensuring the reliability of the model, even when handling large and diverse datasets. Furthermore, assessing the graph’s performance could involve weighting attributes towards institutions with a higher volume of clinical trials. It is important to note that there might be missing stakeholders due to limitations in datasets. These limitations could impact the generalizability of the findings and the model’s performance in other contexts or with more extensive datasets. Moreover, a key limitation of the present work is that the generalizability of this attribute framework is not fully addressed across a broader spectrum of clinical domains, such as acute infectious diseases or fast-evolving oncology trials. These domains present different collaboration dynamics and data characteristics (e.g., highly heterogeneous intervention types or time-sensitive networks) that may require further adaptation of the feature weighting and normalization. Future work will focus on testing the iGraphCTC framework’s robustness by applying the same attribute-fusion methodology to a more diverse clinical trial portfolio. Although our current evaluation demonstrates the superior performance of iGraphCTC, we acknowledge the potential challenges regarding missing or unseen attributes in real-world clinical data. Theoretically, the GCN architecture employed in iGraphCTC mitigates this issue by aggregating features from neighboring nodes, thereby allowing the model to infer missing information through the structural context of the graph. However, to further enhance the robustness of the framework, future research will focus on integrating inductive learning capabilities and imputation techniques specifically designed for incomplete clinical metadata. This will ensure that the predictive performance remains reliable even when certain intervention or geographical signals are unavailable at the time of trial registration.

To address this, further validation can be pursued by integrating data from sources beyond ClinicalTrials.gov, such as the European Union Clinical Trials Register and WHO ICTRP, to create a more comprehensive and globally representative dataset. Moreover, we will expand our future research to include a broader range of baseline models, incorporating both simple network science metrics and advanced semantic pattern analysis. Specifically, we plan to evaluate metrics such as node centrality, clustering coefficients, and shortest paths, and implement path-based prediction models leveraging semantic relationships within our clinical trial data.

The versatility of this model extends beyond its application to the current dataset, demonstrating its adaptability to diverse data sources within and beyond the medical research landscape. Its architecture and methodology lay a robust foundation for implementation across various datasets, facilitating exploration and analysis in the medical domain and other fields. This flexibility indicates its potential applicability in a spectrum of disciplines, enabling the investigation of collaborative networks and relationships across diverse areas of research.

In conclusion, this study represents a significant leap forward in harnessing graph-based models to optimize the efficiency and efficacy of collaborative clinical trials, particularly in the field of diabetes research. Through the introduction of an innovative method for identifying and suggesting potential collaborators, this study not only enriches the scholarly comprehension of collaborative networks but also provides valuable practical tools for stakeholders within the medical research community.

## Data Availability

The materials are publicly available at https://github.com/dxlabskku/iGraphCTC.
